# Real-Time Ichthyoplankton Drift in Northeast Arctic Cod and Norwegian Spring-Spawning Herring

**DOI:** 10.1371/journal.pone.0027367

**Published:** 2011-11-16

**Authors:** Frode B. Vikebø, Bjørn Ådlandsvik, Jon Albretsen, Svein Sundby, Erling Kåre Stenevik, Geir Huse, Einar Svendsen, Trond Kristiansen, Elena Eriksen

**Affiliations:** Institute of Marine Research, Bergen, Norway; University of Hamburg, Germany

## Abstract

**Background:**

Individual-based biophysical larval models, initialized and parameterized by observations, enable numerical investigations of various factors regulating survival of young fish until they recruit into the adult population. Exponentially decreasing numbers in Northeast Arctic cod and Norwegian Spring Spawning herring early changes emphasizes the importance of early life history, when ichthyoplankton exhibit pelagic free drift. However, while most studies are concerned with past recruitment variability it is also important to establish real-time predictions of ichthyoplankton distributions due to the increasing human activity in fish habitats and the need for distribution predictions that could potentially improve field coverage of ichthyoplankton.

**Methodology/Principal Findings:**

A system has been developed for operational simulation of ichthyoplankton distributions. We have coupled a two-day ocean forecasts from the Norwegian Meteorological Institute with an individual-based ichthyoplankton model for Northeast Arctic cod and Norwegian Spring Spawning herring producing daily updated maps of ichthyoplankton distributions. Recent years observed spawning distribution and intensity have been used as input to the model system. The system has been running in an operational mode since 2008. Surveys are expensive and distributions of early stages are therefore only covered once or twice a year. Comparison between model and observations are therefore limited in time. However, the observed and simulated distributions of juvenile fish tend to agree well during early fall. Area-overlap between modeled and observed juveniles September 1^st^ range from 61 to 73%, and 61 to 71% when weighted by concentrations.

**Conclusions/Significance:**

The model system may be used to evaluate the design of ongoing surveys, to quantify the overlap with harmful substances in the ocean after accidental spills, as well as management planning of particular risky operations at sea. The modeled distributions are already utilized during research surveys to estimate coverage success of sampled biota and immediately after spills from ships at sea.

## Introduction

Pelagic drift and environmental exposure of ichthyoplankton (egg, larvae and juvenile fish) of Northeast Arctic (NEA) cod and Norwegian spring-spawning (NSS) herring in relation to variability in recruitment indices (measured as survival until the 0-group stage, i.e. about 5 months old pelagic juveniles) have been the focus of many studies, e.g. [1], [2]. Particular emphasis has been put on early life history of fish due to the high mortality rates experienced by fish during this stage. Several hypothesis suggest key processes impacting survival in early stages of fish, e.g. match-mismatch [3], [4], bigger is better [5], and member-vagrant [6] hypotheses. However, none of these hypotheses, when considered alone, can explain the variability in recruitment in these fish stocks. [7] showed that modeled flow of Atlantic Water to the Barents Sea and modeled local primary production within the Barents Sea, partly representing several of the processes described above, accounted for 70% of the variability in cod with a 3-year lead. Hence, various, synergistic mechanisms may act in different ways to affect the feeding, growth and survival of early life stages as they drift from their spawning grounds along the Norwegian Coast to nursery areas located both along the Norwegian coast (herring) and into the Barents Sea (cod and herring). Along the drift routes the offspring need to find prey and avoid predators. Vertical positioning in the water column is important to the larvae as it affects the interactions with prey and predators and influences the drift routes [1], [8]. Circulation features affecting drift occur on many scales and it is still not clear what horizontal resolution is needed in numerical models to adequately resolve the ichthyoplankton drift routes. However, there seems to be a general agreement that it should at least be on the order of the baroclinic Rossby radius [9], about 5 km along the Norwegian Coast [10], though decreasing northwards with the increase in the Coriolis parameter.

The last decade of improvement in computer technology has enabled high temporal and spatial resolution in biophysical models which provide crucial information on how larval fish might interact with the environment. However, in addition to unveiling historic biophysical links it is also important to develop tools to report real-time distribution and abundance of the ichthyoplankton. Such short-term prediction systems were initially developed for reporting on real-time developments of the physical state of the ocean, e.g. wave-heights, currents and ice-drift, all of which are important for maritime safety. The systems were further developed to also report on biogeochemistry, e.g. algal blooms of critical knowledge to aquaculture. Currently, the European Union funded project MyOcean (www.myocean.eu) aims at integrating European efforts therein by building a pan-European capacity in operational oceanography including major centres involved in operational forecasting and monitoring. A system for operational assessment of ichthyoplankton distribution can be useful in many ways. Firstly, it can be consulted while surveys are ongoing to evaluate and modify the survey design. Secondly, if there are accidental spills of harmful substances in the ocean, an operational larval drift system can immediately report on area overlap with ichthyoplankton. Finally, such a system could be consulted when deciding on time and place for allowing particular risky operations at sea, which could result in increased mortality of ichthyoplankton.

Such a system is now developed in a combined effort between the Institute of Marine Research (IMR) and the Norwegian Meteorological Institute (met.no), where met.no runs a version of the Princeton Ocean Model (MI-POM, [11]) and IMR utilizes the MI-POM two-day forecast to run an individual-based fish larvae model (IBM) for NEA cod and NSS herring. The outcome is numerically processed and made available online either as distribution maps (www.imr.no/larvedrift) or NetCDF files on request. The system has successfully been operating since 2008 and this paper describes the technical details of the model setup, biological constraints, and potential use. Furthermore, we evaluate the results against field observations to indicate the consistency between model predictions and observations of 0-group fish.

## Methods

### 0-group data

The international 0-group fish survey in the Barents Sea is a pelagic juvenile fish survey where the fish species are sampled by the end of the period of pelagic free drift about 5 months after spawning. It has been carried out annually since 1965. In 1980 a standard trawling procedure was recommended by ICES [12], the International Council for the Exploration of the Sea, and has been used on both Norwegian and Russian vessels since then. Since 2003 it has been part of a Joint Norwegian-Russian ecosystem survey in the Barents Sea, designed and jointly carried out by IMR (Norway) and PINRO (Russia). The survey only covers the Barents Sea and therefore not the entire cod and herring 0-group, which can be distributed farther west in the Norwegian Sea and into the fjords along the Norwegian coast.

The standard gear is the “Harstad trawl”, a pelagic trawl with 20 by 20 m mouth opening, 7 panels and a cod end. The panels have mesh sizes varying from 100 mm in the first to 30 mm in the last [13]. The standard trawling procedure consists of predetermined tows at three or more depths, each of 0.5 nautical miles (nm), with the head-line at 0, 20 and 40 m and with a vessel speed of 3 knots. Additional tows at 60 and 80 m, also of 0.5 nm, were made where a dense concentration of fish was recorded deeper than 40 m depth on the echo-sounder.

The computation of abundance indices is made using the stratified sample mean method of swept area estimates [13], [14]. The fish abundance was estimated using only pelagic trawl (0–60 m) catches. For each trawl haul the fish abundance per nm^2^ was calculated based on catch and trawl data (depth intervals, effective opening and distance trawled).

### Numerical ocean model and IBM

The ocean model used is MI-POM (Norwegian Meteorological Institute's version of the Princeton Ocean Model) described in e.g. [11], [15], [16], run operationally at met.no as their core ocean prediction model. The 4 km resolution domain covers the Nordic Seas, the North Sea and the Barents Sea and uses monthly mean climatological boundary conditions from [17] along its open boundaries. Atmospheric forcing is retrieved from met.no's operational Hirlam 8 km model. The heat flux formulations have been adjusted for Norwegian conditions [18] and the model also includes a simple nudging scheme to assimilate satellite SST products. Tides are included and described by the eight harmonic components (M2, S2, N2, K2, Q1, O1, P1 and K1) taken from a barotropic tidal model. The tidal forcing is applied at the lateral edges of the model. A daily forecast of the following two days is routinely made available.

**Figure 1:**
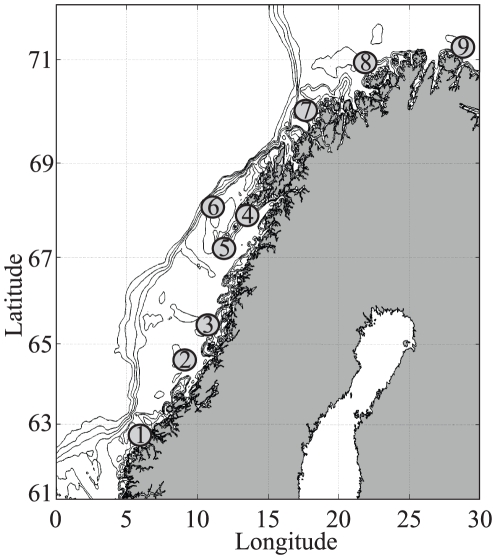
Spawning ground distribution. The spawning grounds used for initializing virtual ichthyoplankton are numbered from 1 to 9. Their relative importance is described in Table 1.

The larval IBM is a particle-tracking model Ladim [19] with a built-in behavioral algorithm for individual larval growth and vertical migration. Ladim reads the daily downloaded ocean forecast (daily averages) and updates the positions of NEA cod and NSS herring larvae using a 4′th order Runge-Kutta advection scheme. Larval growth is temperature dependent for cod with a growth function according to [20], while a fixed daily growth of 0.5 mm/day is applied for herring [21]. The growth affects the swimming capability and thereby the vertical distribution. It is assumed that larvae ascend during night and descend during day because they are visual feeders and dependent on light availability. Observations show that cod larvae are rarely found above 5 m depth and deeper than 40 m [22]. Upper and lower limits are therefore set to 5 and 40 m. A total of about 100 000 particles are initialized at spawning grounds representative for the most recent years during the spawning/hatching season which lasts from March until April [23], [24]. The relative importance of the different spawning grounds (Figure 1) is shown in Table 1.

**Table 1:** Spawning grounds for cod and herring numbered from 1-9 in accordance with Figure 1, and the percentage of all ichthyoplankton initialized at the respective spawning grounds.

Spawning ground	1	2	3	4	5	6	7	8	9
NEA cod (%)	5		5	20	10	20	25	10	5
NSS herring (%)	50	20	10		20				

Particles are counted within each of the 4 by 4 km grid cells before filtering horizontally by convolution with a standard normal distribution covering 5 by 5 cells normalized to conserve mass. This is done to compensate for a relatively low number of particles and to smooth the noisy maps when displaying the results. However, the data behind the maps can also be made available on the web page for downloading. The concentrations are normalized according to the daily maximum value.

All subparts of the system (download the ocean forecast, update particle positions, process particle distribution and abundance into maps and Netcdf files, upload results to a ncWMS server) are written in Fortran or Matlab and integrated by a python script run regularly on an IMR server by a cron job. By maneuvering in the calendar on the web page one can display the distribution map of any day in the pelagic free drift period from March to September during the years 2008 until today.

## Results

**Figure 2:**
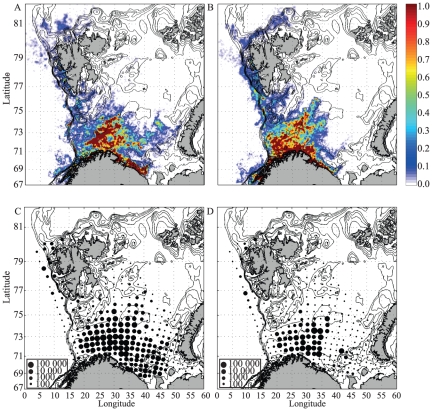
Modeled and observed distributions for NEA cod and NSS herring September 1^st^ 2010. Modeled distributions for NEA cod (A) and NSS herring (B) based on initialization of particles according to spawning grounds location (Figure 1) and relative importance (Table 1) September 1^st^ 2010. Colors indicate abundance relative to maximum abundance for the given time on a logarithmic scale. The corresponding observed distributions for NEA cod (C) and NSS herring (D) where each dot indicates a station on the survey grid and the size of the dot indicate the abundance.

Figure 2 shows an example of modeled distribution maps for NEA cod (A) and NSS herring (B) along with the corresponding observations (C,D). In contrast to field data collected once or twice a year the modeled distributions are available on a daily basis. However, a systematic evaluation of the modeled distributions is necessary to determine their reliability. There is no single test to determine the reliability of the model output, one needs to evaluate both the different sub-models (ocean forecast, particle tracking algorithm and vertical migration scheme) and the final ichthyoplankton distribution. The MI-POM system has been thoroughly validated for the upstream domain of the North Sea ([16] and references therein), the southern part of the spawning grounds [25] and in the core of the drift area [26]. The particle tracking algorithm and the growth and vertical migration scheme has been thoroughly investigated in several studies [1], [2], [19], [20], [21]. We therefore focus on comparing the modeled 0-group distributions in early September with data from IMR surveys at the same time.

**Figure 3:**
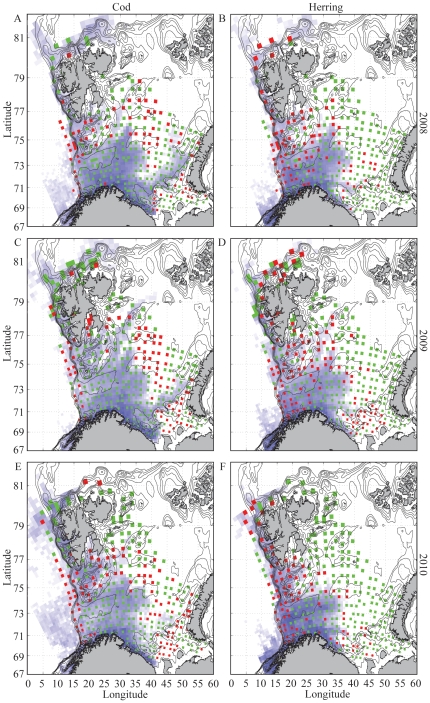
Quantification of percentage area overlap and abundance weighted area overlap between modeled and observed distribution September 1^st^ 2008–2010. Modeled and observed juveniles are interpolated to a 25 by 25 km grid [27] for cod and herring in 2008 (A,B), 2009 (C,D) and 2010 (E,F). Blue cells show concentrations of modeled particles where there are no observations (dark blue indicate relatively higher concentrations than lighter blue). Green cells show that there are observations available and that they are consistent with the model, i.e. either 0-group fish are present in both or in none of them. Red cells show that there are observations available but that they are inconsistent with the model, i.e. either 0-group fish are present in the model but not in observations, or the other way around.

The 4 km model grid resolution is significantly higher than the horizontal sampling grid from the surveys. In order to compare the overlap between modeled and observed juveniles we interpolate both to a 25 by 25 km grid previously defined by [27]. Figure 3 shows the resulting modeled (d_m_) and observed (d_o_) 0-group distributions of NEA cod (A,C,E) and NSS herring (B,D,F) for 2008 (A,B), 2009 (C,D) and 2010 (E,F). Shades of blue indicate cells occupied by modeled particles but where there are no observations. Dark blue indicate relatively higher concentrations than lighter blue. Observations are only available within cells colored by green and red. Green cells show that modeled and observed concentrations are consistent, i.e. either 0-group fish are present in both or in none of them. Red cells show that modeled and observed concentrations are inconsistent, i.e. either 0-group fish are present in the model but not in the observations, or the other way around. We quantify the overlap in percent as the sum of grid cells where either (d_o_, d_m_) >0 or (d_o_, d_m_)  = 0 (i.e. green cells) divided by the total number of grid cells with observations (i.e. green and red cells). Cells where the model indicate juveniles but there are no observations are left out of the overlap estimates, as we cannot determine the quality of the model prediction. The numbers are reported in Table 2. In addition, we partitioned the observed and modeled abundance as either high or low (depending on whether they were above or below median) and weighted the overlap estimate. These results are reported in parenthesis in Table 2.

**Table 2:** Percentage overlap varies between years from 61 to 73%.

Year	2008	2009	2010
NEA cod (%)	73 (66)	73 (67)	69 (71)
NSS herring (%)	68 (64)	61 (63)	61 (61)

Numbers in parenthesis are percentage overlap when weighted with concentrations characterized by above or below the median.

The percentage overlap varies between years from 61 to 73%. When weighted with concentrations, effectively posing a stronger criterion for overlap, the percentage decreases in 2008 for NEA cod and NSS herring and 2009 for NEA cod (Fig.3A,B,C) by 7, 4 and 6% respectively. However, for NSS herring in 2009 and NEA cod in 2010 (Fig.3D,E) the overlap in fact increases by 2%, indicating that the area overlap fits even better when taking concentrations into consideration. The area overlaps are higher for cod than herring in all years. The differences are less when weighted with concentrations in 2008 and 2009, though opposite in 2010.

In general the main features of the modeled distributions tend to compare well with the observations (Fig. 2); i) they are limited in the west by the shelf edge, ii) they are limited by the polar front in the northeastern Barents Sea [28], iii) most particles have been advected into the Barents Sea while some are advected to the west and north of Spitsbergen, and iv) NEA cod are distributed farther east then NSS herring.

## Discussion

### How can the model system be utilized?

This study describes a numerical system used to predict real-time ichthyoplankton distributions of NEA cod and NSS herring based on a two-day ocean forecast by the national meteorological institute of Norway. Although the system has only been running for the previous four years, it has already proven useful in situations with urgent requirement for updated ichthyoplankton distributions. One such application addressed the need to know whether the same larval patches were sampled during two subsequent days. Another application was a sinking vessel, and subsequent fuel spill, at a bank structure in the typical drift paths of ichthyoplankton and the need for a preliminary assessment of possible overlap. However, there are a number of potential benefits of a model system for operational larval drift.

Firstly, a real-time modeling system can be consulted while surveys are ongoing to evaluate and modify the survey design. As there are fundamental limitations to the model predictions we know that it may never be able to represent the ichthyoplankton distribution exactly, but it may still be able to indicate whether the survey is covering the main parts of the distribution. Additionally, it may prove important while conducting dedicated process studies (in addition to the 0-group surveys) when knowledge of day-to-day dispersal may impose restrictions on the validity of a study.

Secondly, potential contamination of marine habitats by accidental spills and long-term introduction of harmful substances motivate the creation of an operational larval drift system that can report on potential overlap with ichthyoplankton in real-time. Also, it may be necessary to prioritize the protection of certain areas due to limitations in resources (e.g. personnel, vessels, chemicals) and a system for real-time surveillance may guide decision-makers in taking the right decisions.

Finally, the system enables monitoring of ichthyoplankton exposure to long-term contamination such as radioactivity, agriculture pollution through freshwater runoff along the coast, and waste-water from petroleum industry. Similarly, the system may be consulted when conducting particularly risky operations at sea where spatiotemporal distribution of ichthyoplankton may be taken into consideration to limit potential risk.

However, while utilizing such a tool it is important to be aware of the limitations. Sources of errors relevant to this study can be divided into those resulting in erroneous model predictions and uncertainties in the field data used for evaluating the model result. The bulk part of the errors in the first category is due to poorly understood processes, such as the motivation for vertical migration in fish and lack of data on prey and predator distributions causing area-specific and time-specific mortality of the offspring. We have therefore tried to keep the model code as simple as possible in order to avoid complex algorithms representing poorly understood processes.

### Sources of errors in the model predictions

We assume that the spawning distribution is similar to the most recent years and that we have a fairly good knowledge of what the spawning distribution has been. A herring larvae survey is conducted in March/April each year to estimate the abundance and distribution of newly hatched larvae. This gives a good indication of the spawning distribution. However, hatching time varies throughout the survey area and hatching may occur after the survey. Also, larvae have been subject to various duration of dispersal dependent on age and may have drifted away from the spawning areas. However, we assume this drift is limited since larvae are mostly caught soon after hatching.

Further, we assume for simplicity that herring eggs hatch at 15 m depth while in nature they are demersal and hatch at the seabed of up to 250 m depth [29]. Insufficient knowledge of the rising velocities is why we have omitted this, though we know it may take some days to ascend to the upper water column after hatching. Contrary, cod eggs have an initial pelagic drift phase of about three weeks where the depth distribution is dependent on the individual egg buoyancy distribution [30]. Algorithms for implementing such a dynamic vertical distribution is described in [31] and utilized in [32], [33] but not yet incorporated here.

Both cod and herring perform a diel vertical migration constrained by an upper and lower boundary and available light at their individual respective depths. Diel migration is here limited by individual length but not dependent on the presence of prey and predators, which is likely to affect vertical habitat selection [1], [8], [34]. The upper and lower boundaries are based on qualitative descriptions from different historic surveys [35], and should be considered approximate as the process of vertical migration is poorly sampled and not well understood.

The ocean forecast by met.no is modeled with MI-POM on a grid with a horizontal resolution of 4 by 4 km. Important small-scale dynamics that will clearly affect dispersal are therefore not included [36]. Because of computational costs it is not possible to both resolve all scales of importance while at the same cover the area of concern. Important effects of this are that dispersal on scales less than about twice the grid resolution is truncated and resulting trajectories are smoother than in reality and that the modeled spread of particles is likely underestimated. Specifically, we see in these particular results that trajectories tend to divert towards shore and overestimate concentrations close to the coast. This might be a result of misrepresentation of sub-grid scale processes or suboptimal handling of coastal boundaries in the particle tracking. In addition, air-sea-wave interactions including Stokes drift are not included in the ocean forecast and are therefore also a source of error in the modeled drift. We are addressing these challenges in ongoing studies.

Finally, natural mortality is not included since spatiotemporal variation therein is one of the main knowledge gaps of early life history in fish. Natural cod egg mortality was studied by [37] and they found that the increased egg mortality in 2001 was connected to age, size and condition of the spawning cod females, and increasing fraction of first time spawners may negatively influence both egg and larval survival. Additionally, both eggs and fish larvae are common prey for several species. Also, studies have revealed indications of spatiotemporal variations in natural survival in ichthyoplankton [38], [39], but details on how this vary through the season are complex and remain a continuous area of research. Our approach is therefore to leave this out completely and acknowledge the limitation this has on the predictive capabilities of the model system.

The present system is run without any kind of data assimilation. Assimilation of data is in principle easy with such a system, as a particle distribution may be modified and the application restarted without much harm to the model dynamics. However, there is little information available. For herring the distribution may only be reinitialized after the larvae survey in March/April, shortly after hatching. For NEA cod no such data is available.

### Sources of errors in the observational data

The 0-group survey has been carried during August-September, and is clearly not synoptic. This has not been accounted for in abundance estimations as drift paths and swimming behavior of the fish during this period are not well known. The capture efficiency of the sampling trawl differs between species and decreases with decreasing 0-group length [40], [41]. Hence, a correction factor was therefore included during the original storage of the data to avoid underestimates in abundance [14]. In addition, the transition in cod from pelagic free drifting to bottom settled is a rather prolonged process occurring gradually in September-October in the Spitsbergen area and October-November in the Southern Barents Sea [42]. This may lead to an underestimate of abundance and distribution as juveniles may escape the sampling volume and an evaluation of the model prediction on false premises. Herring, on the other hand, does not settle to the bottom.

We compared the model predictions for early September with observations of 0-group cod and herring from the same time, though the surveys typically span several weeks. Hence, there is a chance for repeated sampling of a juvenile patch or missing patches as they ‘slip through’ survey masks while we steam for the next sampling station. However, increasing the sampling frequency can compensate for such events, although it is still debated what resolution is required to obtain a representative description of juvenile distribution. One argument is that surveys need to resolve the main physical features of the ocean investigated to ensure a representative estimate of abundance [9], [43]. To a first guess this is likely to be represented by the baroclinic Rossby radius. Whether this is true can be checked by oversampling the area during a few test surveys. The September surveys of cod and herring in the Barents Sea are not close to such sampling rates. Here the distance between stations are more on the order of 25–35 nautical miles.

Survey design is made in advance, but may be modified during the cruise to reach zero-levels of abundance to ensure that the entire distribution is covered. However, this is not always the case, either because there is not enough time to explore the outskirts of the distribution area or because low levels of abundance may falsely be misinterpreted as the boundary. Incomplete coverage will anyhow complicate the evaluation of model predictions, and in this study we have therefore chosen to not let model predictions outside the surveyed area affect the overlap estimate.
